# Spatial Variation in the Mercury Concentration of Muscle Myomeres in Steaks of Farmed Southern Bluefin Tuna

**DOI:** 10.3390/foods4020254

**Published:** 2015-06-16

**Authors:** Kirstin Ross, John Edwards

**Affiliations:** School of the Environment Health and Environment Health Science Building, GPO Box 2100, Adelaide 5001, South Australia; E-Mail: john.edwards@flinders.edu.au

**Keywords:** mercury, lipid, muscular tissues, myomere, spatial variation, southern bluefin tuna, *thunnus maccoyii*

## Abstract

Mercury concentration in the muscular tissue of farmed southern bluefin tuna, *Thunnus maccoyii* (SBT) is known to vary. Data suggests that mercury concentration is negatively correlated with the lipid concentration of tissues. Those areas that accumulate higher levels of lipid are noted to have a lower mercury concentration than lean tissues. Here we further delineate variation in mercury concentration within SBT muscular tissues by determining the concentration of mercury in the muscle myomeres (those sections within whole muscles) of transverse sectional steaks of farmed SBT. Mercury concentration in myomeres is observed to significantly decrease with dorsal and ventral distance from the spine or lateral line of fish. By extension, evidence is provided for the variation of mercury concentration within tissue cuts present in SBT steaks. This paper provides the first documentation of variation in mercury concentration within muscular tissue of fish.

## 1. Introduction

Mercury has long been recognised as a toxic contaminant of fish which binds directly to proteins in the muscular tissues [1,2,3]. Early investigations into the spatial distribution of mercury in fish suggested uniform concentration of mercury throughout the muscular tissues. A pioneering study conducted by Freeman and Horne [4] examined variations in mercury concentration of muscular tissues of swordfish (*Xiphias gladius*) at: (1) intervals longitudinally down the length of fish; (2) intervals along the circumference of transverse sections of fish; and (3) intervals of depth (from the epidermis through to the internal cavity) in transverse sections fish. Results of this study indicated that mercury was uniformly distributed in the muscular tissues. Consequently, it was concluded that a small biopsy of tissue would be representative of whole tissue mercury concentration.

Detection capabilities are likely to have increased since publication of the study by Freeman and Horne [4], which did not include the limit of detection of mercury concentration. However, to date, many studies assume uniform mercury concentration in fish tissues. When determining mercury concentration of fishes it has long been common practice to sample a small subsection of tissue the location of which may vary according to practical and economic convenience [5,6,7,8,9].

**Figure 1:**
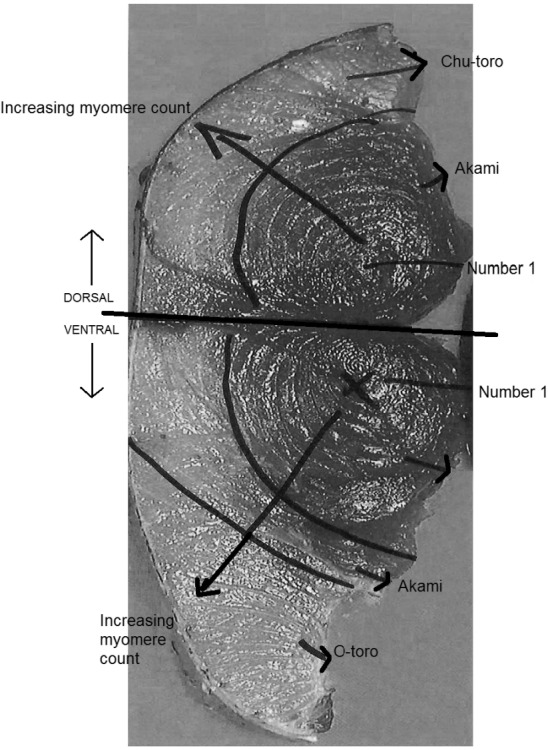
Photograph of southern bluefin tuna, *Thunnus maccoyii* (SBT) transverse sectional steak indicating myomere structure (including myomere number 1) and location of market tissue cuts (akami, chu-toro, o-toro).

While this assumption may be appropriate for many species, bluefin tuna (*Thunnus* spp.) are recognised as having distinct tissue groups. Bluefin tuna muscular tissues are marketed as “akami”, “chu-toro” or “o-toro” and are identifiable based on lipid content, colour, location and muscle structure (see Figure 1).

The lean akami tissues are located at the core of the fish surrounding the dark muscle (not usually consumed) and spinal cord; chu-toro tissues have a medium level of lipid and form a layer between the akami and the skin; and the o-toro tissues have a high lipid content and are located surrounding the stomach cavity [10].

Each of these marketed tissue cuts are reported to have a unique proximate composition [11,12,13,14,15,16] as well as the potential for unique contaminant profiles [10,17].

In a previous study we reported spatial differentiation in the mercury concentration of farmed southern bluefin tuna (*Thunnus maccoyii*) (SBT) muscular tissues. The mercury concentration of composite samples of all edible tissues on farmed SBT and each of the marketed tissue cuts of these fish were found to be inversely correlated to lipid content. Despite differences in fish size, condition and culture history, the relationships between mercury concentration and lipid content was found to be consistent across each fish and tissue type [10]. Lipid accumulation during culture was speculated to dilute mercury associated with fish tissues, and the higher lipid in certain market cuts (e.g., o-toro) over others was speculated to drive cross carcass variation in the mercury concentration of muscular tissues [10,11].

Here we build on the current understanding of the distribution of mercury in SBT tissues by identifying variation in the distribution of mercury within myomeres within each tissue cut. To the best of our knowledge this is the first time such a detailed study on the distribution of mercury within fish tissues has been conducted since the 1973 study on swordfish by Freeman and Horne [4].

## 2. Methods

### 2.1. Experimental Design

Specimens were obtained from commercially stocked and operated experimental farm pontoons. The operational procedure consisted of the purse-seine capture of wild SBT in the Great Australian Bight, Australia in March 2005. Over a period of weeks SBT were towed to the coastal waters of Port Lincoln, where they were transferred into sea pontoons and fattened on a mixture of Australian and imported baitfish species until harvest. Five specimens were harvested in August 2006 after a culture period of 18 months. SBT were harvested and processed according to standard commercial operating techniques, and were received, eviscerated and bled as standard for export-bound product [18]. Samples were placed in single use, unopened medical grade urine sampling containers.

### 2.2. Spatial Variation in Mercury Concentration within Marketed Tissue Cuts

Examination for variations in the mercury concentration within SBT marketed tissue cuts was achieved by sampling transverse sections (or steaks) at intervals of depth from the epidermis through to the internal cavity. Sampling techniques are based on those used by Freeman and Horne [4]. However, rather than sampling arbitrarily defined units of depth, the current study examined variation in the mercury concentration between myomeres of SBT tissue.

The muscles of fish are layered rather than bundled as is the case for other vertebrates. Each layer, or sheet, of muscles is called a myomere and is separated from its neighbour by a sheet of connective tissue [19]. Within SBT steaks myomeres form clear bands of muscle. Each marketed tissue cut within SBT steaks contains multiple myomeres (Figure 1).

### 2.3. Sample Preparation

In the laboratory the fork length (cm) and weight (kg) of specimens were recorded. The head and tail were removed, and the remainder of the carcass was split into left and right halves by slicing all flesh away from the spinal bone and major vertebral bones. Each half carcase was processed separately, either for a composite sample of all edible tissues present or for sampling of SBT steaks.

Composite tissue samples were prepared as described in Balshaw, *et al.* [11] and were representative of all white muscular tissues from one side of a SBT. Although these composite tissue samples were not used for analysis in this study, they were used as an in-house standard for mercury analysis as described in Section 2.4.

In order to sample SBT steaks two vertical cuts were made in SBT half carcases. The first cut was an anterior vertical cut placed immediately posterior to the second dorsal fin and run down to immediately anterior of the anal fin. The second cut was placed at the midpoint between the posterior vertical cut and immediately posterior of the gill arch (the point from where the head was removed). These cuts separated the carcase into three sections. A cross sectional steak approximately 6 cm thick was then removed from each SBT at the midpoint of the centre section. Myomeres were carefully separated away from SBT steaks avoiding the dark muscle and lateral superficial muscle (Figure 1). However, not all myomeres were able to be successfully separated from one another necessitating pooling of myomere samples in some cases. Each individual myomere (or pool of myomeres) were finely diced so that samples could be collected that were a homogenate representative of the whole myomere. Single use scalpels were used for dissection and dicing. All samples were stored in polyethylene plastic containers and stored at −80 °C until analysis.

### 2.4. Mercury Analysis

The total mercury concentration of SBT myomeres was determined by the dissolution of fish tissue with an acid digestion procedure modified from that used by Kim [20]. This consisted of an acid digestion (10 mL of concentrated nitric acid/sulfuric acid mixture, 2:1, v/v) of up to 1.5 g SBT tissue (wet weight) in acid-cleaned glass tubes (i.d.: 25 mm; length: 255 mm), covered with glass beads and heated (70 °C) for a minimum of 6 h. After cooling 10 mL concentrated hydrochloric acid was added and samples were left for up to 24 h until all fizzing had ceased. Digests were diluted with Millipore water (0.5 mL digest: 10 mL water) and mercury was reduced by stannous chloride (1.1% in 1% hydrochloric acid) using a hydrochloric acid carrier solution (3% in Millipore water). Mercury determination was by means of cold vapour atomic absorption spectroscopy (Perkin Elmer Flow Injection Mercury System) using a nitrogen carrier gas. The limit of detection was 0.01 mg/kg.

To account for variation between each analytical run, each run contained all samples pertaining to a single SBT steak along with a standard calibration curve, triplicates of internationally certified standard reference materials dogfish muscle DORM-2 (National Research Council of Canada, Canada) and/or fish tissue IAEA-407 (International Atomic Energy Agency Analytical Quality Control Services, Austria), and an in-house standard reference material. In-house standard reference materials were developed for each individual SBT analysed. These consisted of SBT whole tissue composites described in Section 2.2. A portion of each of the composite tissue samples were sent frozen to an external accredited laboratory (AgriQuality, New Zealand) for analysis. The remaining portion of each composite sample was retained and used as in-house reference material of known mercury concentration, unique to each SBT sampled (*n* = 5). The decision to use SBT composite tissues as reference material was rationalised by the uniquely elevated lipid content of farmed tuna tissues and the use of wet tissue digestion methods for mercury analysis. The high lipid content of SBT tissues, and in particular that of the o-toro tissues [11] may limit the comparability of commercially available reference materials which were comprised of dried fish tissues of unknown lipid content.

### 2.5. Statistical Analysis

Statistical analyses were performed using the statistical package Prism with a significance value of *p* ≤ 0.05. Data were tested for normality using D’Agostino-Pearson normality test. Data were analysed using a repeated measures analysis of variance (ANOVA) in which columns represent SBT (*n* = 5) and rows represent each myomere in SBT steaks. For the purposes of analysis, myomeres were sequentially numbered according to dorsal and ventral distance from the spine or lateral line of SBT–1 is first from the lateral line, 2 is second, 3 is third and so on (Figure 1). Differences in the number of myomeres analysed for mercury in each SBT steak resulted in data being unevenly distributed. Consequently, rows with incomplete data across all 5 SBT were deleted and myomere number became an arbitrary indication of distance from the lateral line. Mercury concentration in myomeres were normalised with myomere 1 representing 100%.

## 3. Results and Discussion

Variation in mercury concentration was found to be statistically significant both between SBT (*F* = 21.7; *p <* 3.28 × 10^−7^), and between myomeres (*F* = 9.7; *p <* 1.0 × 10^−4^). A number of factors may have contributed to produce significant variation between SBT (e.g., fish size, condition factor and age). Differences in the number of myomeres in each SBT resulted in an inability to match myomeres across SBT. As such, myomeres are an arbitrary indication of distance from the lateral line (and spinal cord) and add to the variability in data between SBT. Moreover, although significant variation was detected in mean mercury concentration of SBT myomeres, spatial trends were only apparent in four of the five SBT sampled. Four SBT were noted to decline in mercury concentration with increased distance from myomere 1, however, in the fifth animal, myomere mercury did not decline with distance from myomere 1. This potentially anomalous animal skewed data upwards and increased the variance between SBT.

Notwithstanding this potentially anomalous fish, the data clearly show an overall reduction in mercury concentration with distance from myomere 1 which is located closest to the spine or lateral line. Attainment of a statistically significant difference in the mean mercury concentration between myomeres suggests that systemic changes in mercury concentration of myomeres outweigh variance between SBT.

**Figure 2:**
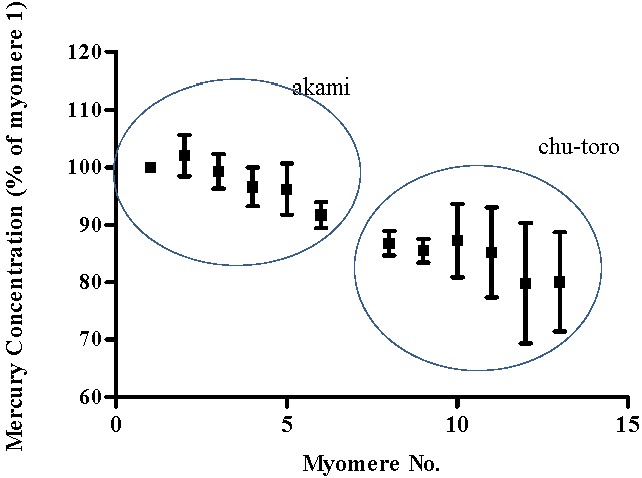
Systemic variation in the mean mercury concentration of muscle myomeres in the dorsal region of SBT steaks. Mercury concentration is presented as a percentage of myomere 1 mercury concentration.

**Figure 3:**
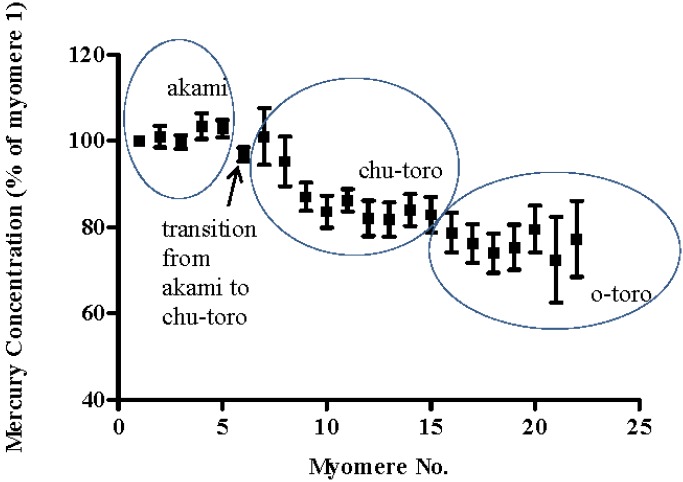
Systemic variation in the mean mercury concentration of muscle myomeres in the ventral region of SBT steaks. Mercury concentration is presented as a percentage of myomere 1 mercury concentration.

In the dorsal region of SBT, a total of 13 myomeres were compared across the 5 SBT. Myomere 1 was found to have a mean mercury concentration of 0.51 mg/kg (±0.03 SE). Mercury concentration of myomeres decreased with distance from the lateral line or spinal cord. Mercury concentration of myomere 13 was 80% (± 8.64 SE) of that of myomere 1 (Figure 2). In the ventral region of SBT, a total of 22 myomeres were compared for mercury concentration. Myomere 1 was found to have a mean mercury concentration of 0.48 mg/kg (±0.06 SE). Again mercury concentration decreased with distance from the lateral line. Mercury concentration of myomere 22 was 77% (± 8.80 SE) of myomere 1 (Figure 3).

The observation that mercury concentration decreases with distance from the spine or lateral line is somewhat consistent with reports on differentiation in the mercury concentration between SBT marketed tissue cuts. Balshaw *et al.* [10] report mean fresh weight mercury concentrations for akami, chu-toro and o-toro tissues of 0.36 ± 0.02 mg/kg, 0.28 ± 0.05 mg/kg and 0.23 ± 0.05 mg/kg respectively. However, the observation that mercury concentration differs between each myomere is novel, and is indicative that variations exist in the mercury concentration within SBT marketed tissue cuts.

Driving mechanisms for this differentiation in the mercury concentration between muscle myomeres remains unknown. Considering that mercury concentration in tissue cuts of wild-capture farmed SBT has been shown to be negatively correlated to lipid content [10], it is possible that variations in the mercury concentration of SBT muscle myomeres reflect variations in lipid deposition. Such a hypothesis is loosely consistent with reports on the functional roles of different muscle groups in tuna activity. Rayner and Keenan [21] report that the regularity of muscle activity in skipjack tuna (*Katsuwonus pelamis*), decreases with distance from the spine. If the same can be said for bluefin tuna species, then it is possible that those muscle myomeres in the outermost regions of SBT tissues that are used less frequently would accumulate higher levels of lipid. However, the current study analysed the mercury concentration of myomeres only and it is unknown if the negative correlation observed between mercury concentration and lipid content of SBT composite tissues and tissue cuts also exist for muscle myomeres.

Future studies may seek to assess the proximate composition and the mercury concentration of muscle myomeres. Such an analysis may further elucidate the role of lipid as a diluting agent on mercury associated with fish tissues as has been suggested in our previous research. However, it is noted that other factors may also be at play. Recognising the important role that blood plays in the distribution of mercury to tissues, it is possible that the ability of tissues to accumulate mercury may be linked to the amount of blood flow to tissue groups. If tissues at a greater distance from the spine are less active, such tissues are likely to receive a reduced blood supply. Additionally, it is possible that less active tissues may contain different types of protein binding sites when compared to active muscular tissues, which may also affect the affinity of mercury for such tissues. The mobility of methyl mercury (the primary form of mercury found in fish muscular tissues) has been attributed to the formation of water soluble complexes that are primarily attached to sulfur atoms of thiol groups such as gluthione [22]. These concepts warrant further investigation, in elucidating the driving mechanisms causing spatial variation in the mercury concentration of fish muscular tissues.

## 4. Conclusions

Mercury concentration is negatively correlated with the lipid concentration of tissues. Those areas that accumulate higher levels of lipid are noted to have a lower mercury concentration than lean tissues. Further delineation shows variation in mercury concentration SBT muscle myomeres (those sections within whole muscles) of transverse sectional steaks of farmed SBT. Mercury concentration in myomeres is observed to significantly decrease with dorsal and ventral distance from the spine or lateral line of fish.
